# How do arbuscular mycorrhizas affect reproductive functional fitness of host plants?

**DOI:** 10.3389/fpls.2022.975488

**Published:** 2022-08-22

**Authors:** Lei Wang, Zhanhui Tang

**Affiliations:** School of Environment, State Environmental Protection Key Laboratory of Wetland Ecology and Vegetation Restoration, Northeast Normal University, Changchun, China

**Keywords:** arbuscular mycorrhizas, sexual reproduction, male fitness, female fitness, nutrient supply, pollen, seeds

## Abstract

Arbuscular mycorrhizal (AM) symbiosis in soil may be directly or indirectly involved in the reproductive process of sexually reproducing plants (seed plants), and affect their reproductive fitness. However, it is not clear how underground AM symbiosis affects plant reproductive function. Here, we reviewed the studies on the effects of AM symbiosis on plant reproductive fitness including both male function (pollen) and female function (seed). AM symbiosis regulates the development and function of plant sexual organs by affecting the nutrient using strategy and participating in the formation of hormone networks and secondary compounds in host plants. The nutrient supply (especially phosphorus supply) of AM symbiosis may be the main factor affecting plant's reproductive function. Moreover, the changes in hormone levels and secondary metabolite content induced by AM symbiosis can also affect host plants reproductive fitness. These effects can occur in pollen formation and transport, pollen tube growth and seed production, and seedling performance. Finally, we discuss other possible effects of AM symbiosis on the male and female functional fitness, and suggest several additional factors that may be involved in the influence of AM symbiosis on the reproductive fitness of host plants. We believe that it is necessary to accurately identify and verify the mechanisms driving the changes of reproductive fitness of host plant in symbiotic networks in the future. A more thorough understanding of the mechanism of AM symbiosis on reproductive function will help to improve our understanding of AM fungus ecological roles and may provide references for improving the productivity of natural and agricultural ecosystems.

## Introduction

The arbuscular mycorrhizal (AM) symbiosis formed between plants and AM fungus is a mutually-beneficial symbiosis prevalent in nature which emerged about 400 million years ago (Selosse et al., 2015). Nearly 80% of vascular plants in terrestrial ecosystems are able to form and maintain such symbiotic relationships with AM fungus (Bhantana et al., 2021). In the symbiotic system formed by plants and AM fungus, plants need to provide the AM symbiosis with carbohydrates produced by photosynthesis, and in return, AM symbiosis has a positive impact on the growth and reproduction of host plants by improving their ability to acquire mineral nutrients (Bhantana et al., 2021). Thus, the cooperative relationship can be maintained stably between AM fungus and plants through mutual help (Bhantana et al., 2021). Although AM symbiosis occurs underground, the regulation of underground AM symbiosis on aboveground growth and development of host plants cannot be ignored. Currently, there are abundant evidences indicated that underground AM symbiosis has directly positive effects on growth and reproductive traits of host plants (Derelle et al., 2015; Bennett and Meek, 2020; Vosnjak et al., 2021; Chen et al., 2022).

**Figure 1:**
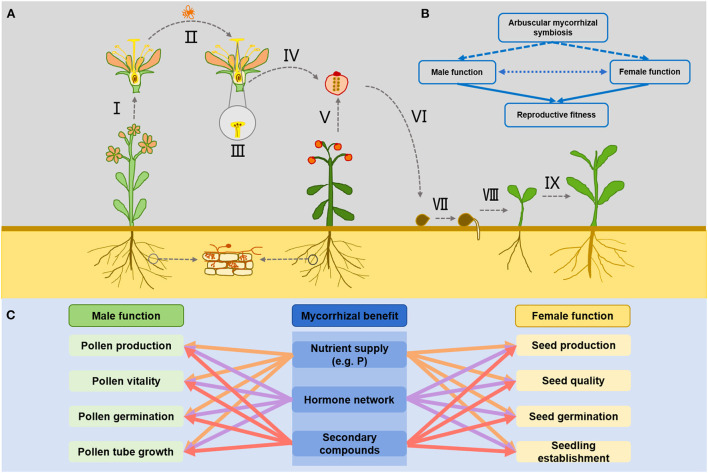
Effects of AM symbiosis on the reproductive function of host plants. **(A)** Represents the way in which AM symbiosis may participate in the realization of male and female functions during plant reproduction, and I represents the pollen production; II represents that changes in floral characteristics may potentially affect pollinators pollination behavior; III represents the pollen germination and pollen tube growth on stigma; IV represents the fruit formation and seed development after successful fertilization; V represents the seeds production; VI represents the seeds quality; VII represents the seeds germination; VIII represents the successful establishment of seedlings after germination; IX represents the growth of offspring seedlings. **(B)** Simplifies the AM symbiosis effects on plant reproduction fitness. AM symbiosis may directly and indirectly affect the male and female functions of plants through nutrient supply and hormone regulation, thus affecting their reproductive fitness. The Mutual adaptation and coordinate between male and female function may also be affected by the AM symbiosis, thus affecting the reproductive fitness of mycorrhizal plants. **(C)** Indicates that AM symbiosis may directly or indirectly regulate the reproductive function of plants through nutrient supply (phosphorus and other essential elements), regulation of hormone network and synthesis of secondary compounds (such as amino acids, proteins, terpenoids, and flavonoids) during the realization of male and female functional fitness of plants.

As a biological factor in soil, AM symbiosis may affect the entire life history of flowering plants involving seed germination, vegetative growth, and sexual reproduction (flowering, pollination, fertilization, fruit set, and seed development) (Figure 1A). Sexual reproduction is an important stage of the plant life history, AM fungus may indirectly affect plant reproductive function through the formation of AM symbiosis with host plants and consequently influence plant population dynamics (Bennett and Meek, 2020). In particular, the effect of AM symbiosis on sexual reproduction fitness of flowering plants should be paid more attention. It is well-known that the sexual reproduction function of plants is manifested in the male and female functions of plants. The individual plants achieve their sexual reproduction fitness through male function, female function, or both depending on the sexual reproductive system of the plants (hermaphrodite/dioecious) (Varga, 2010). Male functional fitness is the ability of pollen production, pollen transfer, pollen germination, and pollen tube growth to fertilize ovules of plants, while female functional fitness is the ability of plants to product mature seeds and the subsequent performance of these seeds (Varga, 2010). The investment of flowering plants in sexual reproduction is influenced by individual nutritional status and environmental factors, especially the presence of AM symbiosis is an important factor regulating the process of sexual reproduction. It has been suggested that the male and female functions of flowering plants may be independently affected by AM symbiosis (Koide and Dickie, 2002) (Figure 1B). At present, it has been widely reported that AM symbiosis are positive relate to reproductive fitness of host plants, and the mechanism that how AM symbiosis affect plant fitness is relatively well-understood. AM symbiosis can assist host plants to successfully achieve reproductive fitness by providing nutrient, regulating hormone balance, and other secondary product production (Stanley et al., 1993; Varga and Kytöviita, 2014; Bennett and Meek, 2020).

It is generally believed that underground AM symbiosis may have direct and indirect effects on male and female functional fitness of host plants. The direct effect is that AM symbiosis promotes the host plant's ability to acquire nutrients and directly drives the host plant to increase resource investment in sexual reproduction. For example, AM symbiosis can improve the uptake and accumulation of major elements (nitrogen, phosphorus, and potassium) and trace elements (zinc, sulfur, copper, iron, calcium, and manganese) in soil by host plants (Chen et al., 2017; Turrini et al., 2018; Bhantana et al., 2021). This positive effect on mineral nutrient uptake directly promotes the growth and development of host plants, and changes the resource acquisition and allocation strategies of host plants, which will make host plants likely to invest more resources in reproductive functions, thereby improve male and female function fitness. For example, increased phosphorus content may have positive effects on flower bud formation and development, flower number, pollen size, and seed production, as it has been shown in the interaction between the non-mycorrhizal root endophytic fungus *Piriformospora indica* and *Cyclamen persicum* (Ghanem et al., 2014). The indirect effects of AM symbiosis on the male and female functions of plants may be that it regulates the synthesis and distribution of secondary compounds in plants by affecting related metabolic pathways and gene expression in host plants (Zouari et al., 2014; Bennett and Meek, 2020). Some studies have shown that AM symbiosis can regulate gene expression and indirectly participate in various metabolic processes of host plants. For example, the functions of photosynthesis, nutrient transport, amino acid synthesis, and terpenoid metabolism were enhanced after mycorrhizal colonization, which undoubtedly affected the growth and development of host plants (Zouari et al., 2014). In particular, AM symbiosis changes the levels of some endogenous hormones (e.g., auxin, gibberellin, etc.) in host plants, which regulate the formation and function of sexual organs (Nuortila et al., 2004; Foo et al., 2013). Thus, AM symbiosis can influence all components of plant sexual reproduction including pollen delivery, pollen germination, pollen tube growth, fruit and seed production, seed germination, etc. by regulating hormones, phenolic compounds, and secondary metabolites production and epigenetic modifications (Varga and Soulsbury, 2017; Cui et al., 2019; Bennett and Meek, 2020; Pons et al., 2020; Ran et al., 2022; Rashidi et al., 2022) (Figure 1C). These results are encouraging us to furtherly confirm that AM symbiosis can have a profound impact on plant reproductive fitness. However, we still lack a deeper understanding of how these two pathways work together. Although it is true that increased nutrient supply or altered hormone levels can affect plant reproductive function from one pathway, but both pathways may coexist in the plant-AM symbiosis system. When multiple regulatory pathways (nutrients, hormones, other gene products, etc.) exist together, does one pathway get overridden by the other or do these effects regulate different aspects individually or do they all work together? That's still unclear to us.

Most flowering plants can produce offspring through sexual reproduction (Hiscock, 2011). AM symbiosis may be involved in various stages of the realization of reproductive fitness of host plants. The changes of male and female functional traits of host plant in the presence of soil AM symbiosis were focused on in this paper. Understanding the trade-off strategy between vegetative growth and sexual reproduction fitness in plants will be a meaningful reference for future efforts to use AM symbiosis to enhance plant productivity. Therefore, we reviewed the relevant researches on how soil AM symbiosis affect male and female fitness during sexual reproduction of host plants. We aimed to understand whether the promotion of host plant traits by AM symbiosis leads to changes in male and female functional fitness. Finally, we propose future research directions that will help to expand existing research area and enable us to more fully understand the feedback mechanism of plant sexual reproduction fitness to belowground AM symbiosis.

## Effects of arbuscular mycorrhizal symbiosis on male functional fitness

### Effect on pollen production and viability

It has been reported that AM symbiosis can affect pollen quantity and quality of host plants (Bennett and Meek, 2020). Pollen production can be affected by AM fungus colonization at three different levels: at the level of flower production, of anthers per flower and pollen production per anther (Varga and Kytöviita, 2014). Because pollen production depends on the availability of resources during pollen development, which may be one of the main reasons why AM symbiosis can have beneficial effects on pollen production and performance (Poulton et al., 2002; Bennett and Meek, 2020). Phosphorus as an essential element for pollen production, is directly involved in the formation of nutrient storage compound (such as phytate) in pollen, which will be metabolized and utilized during pollen tube growth (Varga, 2010; Pereyra et al., 2019). Previous studies have shown that the effect of AM symbiosis on pollen production and performance is mainly due to the improvement of plant phosphorus acquisition, and that AM Fungus inoculation and high soil phosphorus conditions have similar beneficial effects on male function (Varga, 2010). AM fungus can transport phosphorus from the soil to the plant roots through extraradical hyphae, especially during florescence, the abundant phosphorus supply may promote pollen production and viability (Pendleton, 2000; Poulton et al., 2002; Barber and Soper Gorden, 2015; Pereyra et al., 2019). This can be considered as an important way for AM symbiosis to affect male function of host plants (Figure 1C).

AM symbiosis can also affect pollen production and viability by regulating hormone and secondary metabolite content in host plants (Halo et al., 2020) (Figure 1C). It is well-known that plant hormones such as gibberellins (GAs) and jasmonic acid (JA) play important regulatory roles in pollen development (Marciniak and Przedniczek, 2019; He et al., 2021; Amanda et al., 2022). Genes involved in the metabolism of plant hormones and secondary terpenoids metabolism have a systematic response to AM symbiosis. For example, the expression of genes related to the terpenoid synthase (*TPS*) family is up-regulated in the presence of arbuscular mycorrhizal (Zouari et al., 2014). Other studies showed that the expression of auxin-dependent reporter DR5-GUS in AM fungus colonized root cells was higher than in surrounding cells (Etemadi et al., 2014). These results suggested that AM symbiosis can promote the synthesis of auxin (IAA), Gas and cytokinin (CTK). In addition, AM fungus itself may release CTK and IAA and then transport them into the root tissues of host plants, which may also increase CTK and IAA content in plants (Pons et al., 2020). These results suggest that the enhancement of plant hormone synthesis induced by AM symbiosis may play an important role in promoting pollen formation.

Furthermore, AM symbiosis also can affect other secondary compounds in pollen (Figure 1C). For example, AM fungus inoculation has a significant effect on the alkaloid content (anabasine and nicotine) in pollen, which may affect pollen transmission process by pollinators (Davis et al., 2019). Mycorrhizal symbiosis also affects phenolic compounds (such as flavonoids) in plants, and generally increases flavonoid synthesis and accumulation in roots, which regulates IAA transport and signal transduction to promote plant growth (Cui et al., 2019). In AM symbiosis system, flavonoids in turn can participate in mycorrhizal growth and branching, and further promote AM fungus symbiosis with plant (Nascimento and Tattini, 2022). It was found that these flavonoids also have been shown to promote pollen production (Cheyniera et al., 2013). It is worth mentioning that silencing the expression of sucrose transporter *SlSUT2* in plants reduced pollen viability and germination, and then caused male sterility of flowers which could be alleviated by Brassinosteroids (BRs) (Hansch et al., 2020). It was been found that AM symbiosis can increase the content of BRs in roots (Sun et al., 2020). Therefore, these secondary compounds may also have positive effects on male fitness of AM symbiotic plants.

The improvement of pollen quantity and quality reflects the enhancement of male function of plants, because high-quality pollen as male gamete is more conducive to seed formation and development (Ghanem et al., 2014). Generally, AM symbiosis can stimulate pollen formation, increase pollen quantity, and improve pollen quality and viability by improving the ability of plants to obtain nutrients (especially phosphate) from soil and mediating other metabolic pathways (Poulton et al., 2002). High quality and viability pollen are conducive to pollen transfer and fertilization, and determines the successful realization of male function of the host plant (Varga, 2010; Ghanem et al., 2014).

### Effect on pollen germination and pollen tube growth

Pollen is transferred by pollinators to the stigma, where it rehydrates and germinates to form pollen tubes. The pollen tubes subsequently penetrate the stigma and grow through the style toward the ovules in the ovary. After penetration of the embryo sac in the ovule, the sperm cells are released and fertilization takes place (Pezzotti et al., 2002). There are many complicated steps involved in this process. The pistil delivers many compounds to the pollen, such as myo-inositol, flavonoids, proteins, water, and lipids after pollen deposition on the stigma, which maintain the hydration and germination of pollen and the growth of pollen tubes on the stigma (Pezzotti et al., 2002). At the same time, phytate stored in pollen is hydrolyzed into phosphate and inositol, which are used in the synthesis of cell walls and membranes of pollen tube (Poulton et al., 2002). So, nutrients stored in pollen also affect pollen germination, pollen tube growth rate and competition ability with other pollens (Koide and Dickie, 2002). This suggests that we can consider the influence of AM symbiosis on pollen germination and pollen tube growth from the perspective of pollen's own nutritional status and stigma's active transport of nutrients to pollen.

AM symbiosis changes the mineral nutrient supply level of host plants, which may be directly or indirectly affect pollen viability and germination (Poulton et al., 2002; Bennett and Meek, 2020). The nutrient accumulation increase in pollen may be the direct cause of improved pollen germination (Figure 1C). AM symbiosis also promotes the uptake and accumulation of trace elements (boron, calcium, magnesium, sulfur, strontium, etc.) in host plants (Ramírez-Flores et al., 2019). For example, boron (B) is an essential trace element in flowering plants and participates in pollen germination. AM symbiosis may contribute to the maintenance of B homeostasis, increase the passive transport of B and promote the uptake of B in soil by enhancing water transport by aquaporin, thus affecting the pollen germination (Quiroga et al., 2020). Therefore, the importance of AM symbiosis's supply of mineral nutrients in the successful realization of male function of host plants needs special emphasis (Figure 1C). At present, studies on the direct effects of AM fungus colonization on pollen germination are scarce. However, we hypothesized that the benefits of nutrient supply by AM symbiosis during pollen production process might be an enhancement mechanism for pollen germination and pollen tube growth.

The growth of pollen tubes after pollen germination to ovules through stigma is a complex biochemical process involving cell wall digestion. AM fungus colonization affected the synthesis and accumulation of hormones, flavonoids, and mineral elements in plants, which may directly affect their accumulation in pollen during pollen formation. Pollen tube growth may be influenced by the substances contained in pollen and the substances transported by stigma during pollen tube growth. For example, pollen tube growth can be enhanced with the participation of flavonoids and gibberellins (Singh et al., 2002; Taylor and Grotewold, 2005). Trace elements (such as zinc) are also important for pollen tube formation (Bhantana et al., 2021). AM symbiosis usually shows a positive effect on the production and accumulation of these above substances, which is conducive to improving the pollen tube growth (Poulton et al., 2001). Thus, AM symbiosis may enhance pollen tube growth by increasing the content of these substances in pollen and stigma (Figure 1A).

Moreover, the process of pollen tube growth into ovules also need to use expansins to catalyze cell wall relaxation without damaging the cell wall (Cosgrove, 2017; Bennett and Meek, 2020). Expansins have been proved to be a regulatory substance affecting pollen tube growth (Mohanty et al., 2018). Improved AM fungus colonization can induce an increase in the transcription level of expansins (Dermatsev et al., 2010). In addition, calcium (Ca^2+^) also plays an important role in flowering plant (angiosperm) sexual reproduction. Ca^2+^ signals are present during pollen germination and interaction with papillae cells of the stigma surface, during pollen tube growth within the stigma of female flowers, and during sperm release when the pollen tube reaches the ovule (Chen et al., 2015). AM fungus colonization can activate Ca^2+^ channels and induce a series of gene expression, leading to calcium surge in root epidermal cells (Caroline and Martin, 2013). It has been found that AM symbiosis and the increase of Ca^2+^ content synergistically promote the content of gibberellin and flavonoids in roots (Cui et al., 2019). This may increase AM fungus colonization, and promote pollen tube growth in stigma after pollination. Thus, AM symbiosis may affect pollen tube growth by regulating expansins production or altering Ca^2+^ signaling, or by increasing overall pollen viability (Bennett and Meek, 2020). However, how the regulation of AM symbiosis on expansins and Ca^2+^ signals affects the growth of pollen tube after pollination still needs to be further proved by experiments.

## Effects of arbuscular mycorrhizal symbiosis on female functional fitness

### Effect on seeds production

The seeds of angiosperms are developed from the fertilized ovules in the ovary, which can be regarded as the realization of female function (Koide and Dickie, 2002). Plant seed production is closely related to the number of flowers, fruits, and seeds in each fruit (Varga, 2010). Seed production is similar to pollen production and mainly depends on resource availability (Varga, 2010). Therefore, the effect of AM fungus colonization on seed production can be reflected in three aspects: ovule number and viability, seed setting (ratio of mature fruits to flowers), and seed quality.

In addition to ovule number and viability, pollen viability and fertilization capacity are also important determinants of seed production. The effects of AM symbiosis on ovule formation, pollen quality, or successful fertilization were equally important for seed production. Previous studies have shown that AM fungus colonization can significantly increase the phosphorus content in plants, and sufficient phosphorus supply can promote the development of more and better ovules and improve the ovules viability (Poulton et al., 2002; Ghanem et al., 2014). While promoting pollen viability, AM symbiosis also improves ovule viability, and the joint enhancement of pollen and ovule viability ultimately benefits seed production (Poulton et al., 2002). Although one study showed that AM fungus colonization appeared to have no significant effect on ovule production, it could increase pollen production (Philip et al., 2001). So, the successful transfer of large amounts of pollen could improve the fertilization success of cross-pollinated plants, thereby increasing seed production.

The increase of concentrations of photosynthetic products, plant hormones (such as auxin) and the content of essential trace elements after AM fungus colonization will lead to increased flower and fruit numbers (Bona et al., 2017; Saini et al., 2019) (Figure 1A). When fertilization capacity of plants is not affected by AM symbiosis, the increase of flower number and fruit number may directly increase seed yield, but we still need to consider whether AM symbiosis will reduce or not affect fruit abortion. Current researches indicated that AM symbiosis has a positive effect on fruit development and yield (Berta et al., 2014; Igiehona et al., 2021). AM symbiosis enhances the ability of host plant to obtain resources, and increases the resource investment of host plants to sexual reproduction. Under the condition of AM symbiosis, the increase of photosynthetic products, plant hormones (such as auxin), and absorbed trace elements (such as inducing the expression of potassium transporter gene in host plant cell and promoting the uptake of potassium by the host plant) may promote the increase of investment to reproductive organs, thus increasing the development and growth of plant flowers and fruit. The increase in fruit yield also leads to the increase in seed yield (Bona et al., 2015, 2017; Liu et al., 2019). However, the positive effect of AM symbiosis on fruit production may only reflect in the early stages of fruit production, but mycorrhizal can prolong the period of fruit abortion and reduced fruit yield at later stage of the fruit production. This may reflect the higher carbon costs of plant symbiosis with AM fungus at later growth stage, and lead to the decline of potential early benefits of AM symbiosis to total fruit yield (Trimble and Knowles, 1995).

Seed traits in fruits are also very important because seed quality has an important impact on plant reproductive success. The effects of AM symbiosis on seed yield and performance are also largely related to improved phosphorus uptake (Varga, 2010). AM fungus colonization of plant roots can increase the content of nitrogen and phosphorus in the aboveground parts of plants, leading to higher seed yield and quality (Thioub et al., 2019). The resource investment of plants in reproduction may also be regulated by AM symbiosis, and the effects of AM symbiosis on plant hormones and other metabolism products should not be ignored (Varga, 2010) (Figure 1C). AM symbiosis may play a role in the regulation of hormone networks during seed development, such as gibberellin, which is necessary for normal development of seed (Singh et al., 2002). The ultimate effect of AM symbiosis on seeds can be reflected in the changes of mature seeds size and weight. At present, most studies about the effect of AM symbiosis on plant seeds have revealed nutrient content in seeds can be regulated by AM symbiosis. For example, starch, fat, protein, and trace element (zinc) content in seeds and seed weight were significantly increased after AM fungus colonization (Berta et al., 2014; Al Mutairi et al., 2020; Marro et al., 2020; Copetta et al., 2021; Igiehona et al., 2021; Wang et al., 2022a). These positive effects can be attributed to the improvement of nutrient status of host plants (phosphorus, nitrogen, etc.) by AM symbiosis, as well as changes of regulation mechanism mediated by AM symbiosis may also be involved. Transcriptome analysis by RNA-Seq showed that the genes of vacuolar invertase (TIV1) and cell wall invertase (LIN6) synthesis were up-regulated in tomato after AM fungus colonization. Vacuolar and cell wall invertase can cleave sucrose transported from the source organ (leaf) into hexose (glucose and fructose) as a direct source of carbon and energy (Zouari et al., 2014). This pathway may also increase nutrient accumulation (sugar) in seeds and improve seed quality.

### Effects on seed germination and seedlings performance

Seed germination and seedling establishment are also necessary for the successful realization of female function after seed formation (Figures 1B,C). AM symbiosis may have direct and indirect effects on seed germination and seedling establishment of host plants. Direct effects include increasing seed size or nutrients stored in endosperm, and indirect effects include that changing the trait plasticity of parental plant, which may lead to transgenerational transmission of phenotypic plasticity in functional adaptation (Herman and Sultan, 2011; Varga et al., 2013; Varga and Kytöviita, 2014; Yin et al., 2019). It is noteworthy that the transgenerational effect can operate *via* two mutually non-exclusive mechanisms, seed provisioning (seed size, seed nutritional quality, or hormonal balance), and environmentally induced heritable epigenetic modifications in offspring (Puy et al., 2022). Here, we review the effects of AM symbiosis on female functional fitness of host plants from two aspects: the effects of AM symbiosis on seed germination and the growth performance of germinated seedlings.

Seeds produced by plants after AM fungus colonization generally have better germination performance (Bennett and Meek, 2020). It is mainly reflected in the promotion of seed germination and radicle elongation (Koide et al., 1994; Copetta et al., 2021). It is generally believed that AM symbiosis promotes the host plant to devote more resources into seed production during seed formation, which makes the produced seeds easier to germinate (Zhu and Smith, 2001). As mentioned above, when AM symbiosis promotes seed formation, plant hormones (gibberellin, cytokinin, etc.) are also be more allocated and stored in seeds, thus affecting seed germination (Figure 1C). Strigolactones (SLs) (carotenoid derivatives) also participate in the germination process of seed. AM fungus colonization increases SLs (GR24) the synthesis and SLs accumulation of SLs (GR24) in seeds, thus which can improving improve seed germination (Mohanty et al., 2018; Faizan et al., 2020; Rehman et al., 2021). In a recent study on the role of alternative oxidase (AOX) and sucrose in seed germination, it was found that the increase of intracellular sucrose content was not conducive to seed germination, but seeds significantly alleviated sucrose stress and effectively restored normal respiration during germination and then increase germination percentage after AM fungus inoculation (Bharadwaj et al., 2021). Besides, a study has shown that seed DNA methylation was promoted after host plants symbiosis with AM fungus, which is also conducive to seed germination (Varga and Soulsbury, 2017). Therefore, AM symbiosis may improve increase seed germination percentage rate by improving seed performance. This reminds us that the influence of AM symbiosis on plants may not only exist in the plants symbiosis with AM fungus, but also may persist in the propagule (seed).

The seedlings growth performance of seeds after seed germination can be regarded as the true embodiment indicator of the successful reproductive function success and improved adaptability of plants (Bennett and Meek, 2020). If parental plants with AM fungus colonization provide more resource to seed, it can theoretically affect the performance of seedlings germinated from the seeds, and better growth performance of seedlings may be related to increased phosphorus content in seeds (Zhu and Smith, 2001; Varga, 2010) (Figure 1A). Currently, most studies have shown that the growth performance of the next generation seedlings of plants with AM symbiosis is better than that of plants without AM fungus colonization, which can be attributed to the differences of seed quality between the plants with AM symbiosis and without AM symbiosis (Nuortila et al., 2004). One explanation for the improvement of AM symbiosis to next generation seedling growth is that pollen from mycorrhizal plants has higher competition ability than non-mycorrhizal plants. The pollen competition hypothesis predicts that when the number of pollen grains deposited onto stigmas exceeds the number of ovules, natural selection will play a role during the pollination and fertilization process. Moreover, pollen competition has been proved to improve seed fitness, to produce seeds with higher germination percentage and increase seedling growth performance (Varga et al., 2013). The effect of pollen competition on seedling growth performance may become obvious only at later stages of growth and when it was assessed in different resource environments (Kalla and Ashman, 2002; Varga et al., 2013). It is not comprehensive to only consider that increased nutrient content in seeds has a positive effect on seedling growth, since nutrients in the endosperm are quickly consumed as seedlings grow, and this beneficial effect may only occur in the early stages of seedling growth. Therefore, the passive plasticity of parental AM symbiosis on nutrient uptake capacity might be transmitted to the seedling and affect their nutrient uptake capacity, thus improving the growth performance of seedlings (Varga et al., 2013). At present, a study has shown that the characteristics of increased root phosphatase activity of parents after AM fungus colonization can be passed on to seedling, so that the root phosphatase activity of seedling is also improved (Koide and Lu, 1995). This may be an important reason for the improvement of seedling growth. Furthermore, some studies have shown that the epigenetic modification induced by plants to adapt to the environment not only exists in the present generation, but also can transfer “memory” to the offspring (Boyko and Kovalchuk, 2011; Cicatelli et al., 2014). The AM symbiosis induces the changes of epigenetic modifications in host plant, which are transmitted to offspring through transgenerational effects and showing beneficial phenotype in offspring. This may be another reason why offspring are more vigorous and fecundity in same environment. Thus, AM symbiosis may contribute to the overall fitness of a host plant and strongly influence long-term plant population dynamics (Stanley et al., 1993).

## Future directions

Although the effects of AM symbiosis on male and female functional fitness of host plants have been discussed from a positive perspective, reproductive traits of plants are also dependent on resource availability, and AM's ecological function may also be affected by soil conditions (Wang et al., 2022b). The function of arbuscular mycorrhiza varies among AM fungus species and plant genotypes, and was determined by the costs and benefits of mycorrhiza-plant interactions (Bennett and Groten, 2022; Wang et al., 2022b). Therefore, we can predict that the plant reproductive traits could be positively, negatively, or neutrally affected by AM symbiosis, thus the male and female functions may be enhanced, weakened or unaffected. The existing evidences do not support the universality of relationship between AM symbiosis and the host plants with different nutrition uptake strategies, and they can only represent the results of symbiosis in the most suitable state between plants and AM fungus (Grilli et al., 2013; Bennett and Meek, 2020). Further consideration should be given to the differences in environment, characteristics of host plants, and functional compatibility between AM fungus and plant in future.

For the successful realization of reproductive function fitness of plants, it is one-sided to think that AM symbiosis alone enhances male or female function. We also need to fully consider whether AM symbiosis affects the behavior of pollinators during pollen transfer process in cross-pollinating plants (Figures 1A,B). Pollinator activity is an important determinant to seed formation in insect-pollinated plants (Varga and Kytöviita, 2010). The role of AM symbiosis in pollen transfer process has been well-reported (Bennett and Meek, 2020). AM symbiosis can affect the pollen dispersed by pollinators through influencing the number of flowers, nectar content, and volatile organic compounds production (VOCs). The visit frequency and pollination success of pollinator may increase with the increase in contents of nectar, sugar, and amino acids (Barber and Soper Gorden, 2015). Therefore, for cross-pollinating plants, the influence of AM symbiosis on pollinator attraction still needs to be explored. More experiments are needed to directly verify pollinators' response to AM symbiosis, which is particularly important for understanding the successful realization of male and female functions of mycorrhizal plants.

In addition, AM symbiosis may play different roles in reproduction process for seed plants with different sexual systems. Studies have shown that females devote more resources to sexual reproduction than males in dioecious plants, because the reproductive success of females often costs more resources than that of males (Mizuki et al., 2005; Varga et al., 2013). Other studies suggest that AM symbiosis may preferentially promote male functions rather than female functions. In particular, AM symbiosis may improve male functions by enhancing pollen number and size and may decrease female functions by reducing seed yield (Pendleton, 2000; Varga and Kytöviita, 2010; Barber and Soper Gorden, 2015; Bennett and Meek, 2020). In gynodioecious populations, females must compensate for not contributing genes through pollen, as hermaphrodites do, in order to be maintained in the same population (Varga et al., 2013). In fact, pollinators are more likely to visit hermaphrodite flowers, which may be due to the superior number of flowers or floral reward (nectar) to dioecious flowers (Varga and Kytöviita, 2010). So, we need to consider the differences of plant sexual systems when recognizing the influence of AM symbiosis on reproductive fitness. For plants with monoecious, dioecious, or other complex sexual systems, AM symbiosis may regulate their male or female functions by influencing their resource allocation patterns.

In the field environment, the interactions between plant reproduction and the surrounding organisms is more complex. Reproduction is not only promoted by underground symbionts and pollinators, but also affected by a variety of other soil biological factors, including nitrogen-fixing bacteria of leguminous plants and other beneficial bacteria, root-knot nematodes, as well as the herbivores. AM symbiosis can induce immune defense responses of host plants, thus helping host plants resist the infection of multiple plant pathogens and the feeding of herbivores (Hol and Cook, 2005; Wang et al., 2019; Ralmi et al., 2021; Frew et al., 2022). However, it is still not clear how plants in complex ecosystems use AM symbiosis to respond to changes in a wild range of biological and abiotic factors, including interactions with other plants and insects. Plant reproduction may be negatively affected by other stresses, while AM symbiosis and its interaction with other microorganisms may alleviate or enhance these adverse effects. Further, AM fungus can also combine with other beneficial microorganisms [plant growth promoting rhizobacteria (PGPR)] to affect the growth and reproduction process of host plants (Turrini et al., 2018; Mohanty et al., 2021; Rai et al., 2021). Generally, the same as AM fungus, rhizosphere microbes can assist the growth and development of plants by enhancing nutrient uptake, regulating the expression of genes and producing hormones (IAA, CTK, and GAs, etc.) (Dodd et al., 2010; Gao et al., 2022; Yu et al., 2022). PGPR also modulated the formation and development of plant reproductive organs through hormone homeostasis (Sharma et al., 2022). Therefore, PGPR community is also involved in the realization of plant reproductive fitness in rhizosphere. Although PGPR treatment had positive feature on seed germination and seedling growth (Mitra et al., 2021), the effect of vertical transmission of PGPR on plant progeny remains unknown. It is worth noting that some PGPR can promote plant growth better than AM fungus under environmental stress (Durán et al., 2016). That will also be an interesting challenge to better distinguish and quantify the individual contributions of AM fungus and PGPR when combined. In the future, the ecological role of AM symbiosis in plant reproduction under the coexistence of multiple factors will enable us to better understand the influence of AM symbiosis on the fitness of plant male and female function.

## Conclusion

In conclusion, we reviewed the possible mechanisms and pathways of arbuscular mycorrhizal symbiosis' effects on plant reproductive fitness from the two aspects: the direct or indirect effects of arbuscular mycorrhizal symbiosis on male function (pollen) and female function (seed) of host plants. With the development of genomics and transcriptomics, the future application of various components analysis and protein-hormone interaction methods in plants will help us better understand the ecological functions of arbuscular mycorrhizal symbiosis on plant reproduction. At the same time, combining molecular biological and ecological methods to explore the ecological effects of arbuscular mycorrhizal fungus will allow us to better understand the interaction mechanisms between plants and arbuscular mycorrhizal fungus. The combined application of above-mentioned research methods will make us clearer how arbuscular mycorrhizal symbiosis manipulate the changes in plant reproductive fitness.

## Data availability statement

The original contributions presented in the study are included in the article/supplementary material, further inquiries can be directed to the corresponding author.

## Author contributions

LW: data curation, visualization, writing—original draft, writing—review, and editing. ZT: funding acquisition, supervision, resources, review, and editing. All authors contributed to the article and approved the submitted version.

## Funding

This study was funded by the National Natural Science Foundation of China (Grant Nos. 31470446 and 31960228).

## Conflict of interest

The authors declare that the research was conducted in the absence of any commercial or financial relationships that could be construed as a potential conflict of interest.

## Publisher's note

All claims expressed in this article are solely those of the authors and do not necessarily represent those of their affiliated organizations, or those of the publisher, the editors and the reviewers. Any product that may be evaluated in this article, or claim that may be made by its manufacturer, is not guaranteed or endorsed by the publisher.
